# Comparing genome-wide significant and chemosensory variants as instruments for dietary patterns in Mendelian randomization

**DOI:** 10.1093/ije/dyag186

**Published:** 2026-09-09

**Authors:** Phoebe S C Hui, Brooke L Devlin, David M Evans, Liang-Dar Hwang

**Affiliations:** Institute for Molecular Bioscience, The University of Queensland, Brisbane, Australia; School of Human Movement and Nutrition Sciences, The University of Queensland, Brisbane, Australia; Institute for Molecular Bioscience, The University of Queensland, Brisbane, Australia; Frazer Institute, The University of Queensland, Brisbane, Australia; Medical Research Council Integrative Epidemiology Unit, University of Bristol, Bristol, United Kingdom; Institute for Molecular Bioscience, The University of Queensland, Brisbane, Australia; Monell Chemical Senses Center, Philadelphia, PA, United States

**Keywords:** Mendelian randomization, dietary patterns, nutritional epidemiology, cardiometabolic health

## Abstract

**Background:**

Diet is a modifiable risk factor for cardiometabolic disease, yet establishing causality remains challenging. Mendelian randomization (MR) leverages genetic variants as instrumental variables (IVs) to enable causal inference.

**Method:**

Using two-sample MR, we assessed the causal effects of four principal component-derived dietary patterns (DPs)—Unhealthy, Healthy, Meat-based, Pescatarian—on cardiometabolic outcomes including body mass index, coronary artery disease, blood lipids, blood pressures, type 2 diabetes, fasting glucose and insulin, and glycated haemoglobin. Two sets of IVs were employed: conventional genome-wide significant variants associated with each DP, filtered for pleiotropy and directionality; and biologically informed variants in chemosensory receptor genes, given the role of taste and smell perception in food choice.

**Results:**

Using conventional IVs, the Pescatarian DP was associated with reduced fasting insulin (***β***_IVW_ = −0.10 pmol/L per SD increase in the Pescatarian DP score, 95% confidence interval −0.15, −0.04; *P *= 1.19 × 10^−3^), surviving multiple sensitivity analyses. Associations between the Unhealthy DP and elevated blood pressure and glycated haemoglobin should be interpreted cautiously; one of the two filtered IVs was strongly associated with caffeine intake, limiting the attribution of these findings to the DP itself. Chemosensory Receptor IVs yielded null findings, reflecting insufficient power.

**Conclusion:**

Evidence for causal effects of DPs on cardiometabolic traits was limited, with the strongest support for a protective effect of the Pescatarian DP on fasting insulin. Chemosensory IVs demonstrated limited utility for DPs, likely reflecting the heterogeneous and complex sensory profiles of overall diets. Future efforts should consider guideline-based dietary indices to facilitate interpretability and translation.

Key Messages

- We used Mendelian randomization (MR) to evaluate the causal effects of dietary patterns (DPs) on cardiometabolic health and explored whether variants in chemosensory receptor genes could improve instrument specificity given their mechanistic link to food choice.
- A Pescatarian-like DP was associated with lower fasting insulin, whereas evidence for causal effects across other DP–cardiometabolic outcome associations was limited, suggesting that DPs have, at most, modest direct effects on cardiometabolic health when isolated from non-dietary factors in MR.
- Chemosensory receptor variants showed fewer associations with confounding pathways compared with variants selected solely based on genome-wide significance, but explained insufficient variance in composite DPs to support well-powered MR analyses, highlighting the difficulty in identifying strong, diet-specific genetic instruments.

## Background

**Figure 1:**
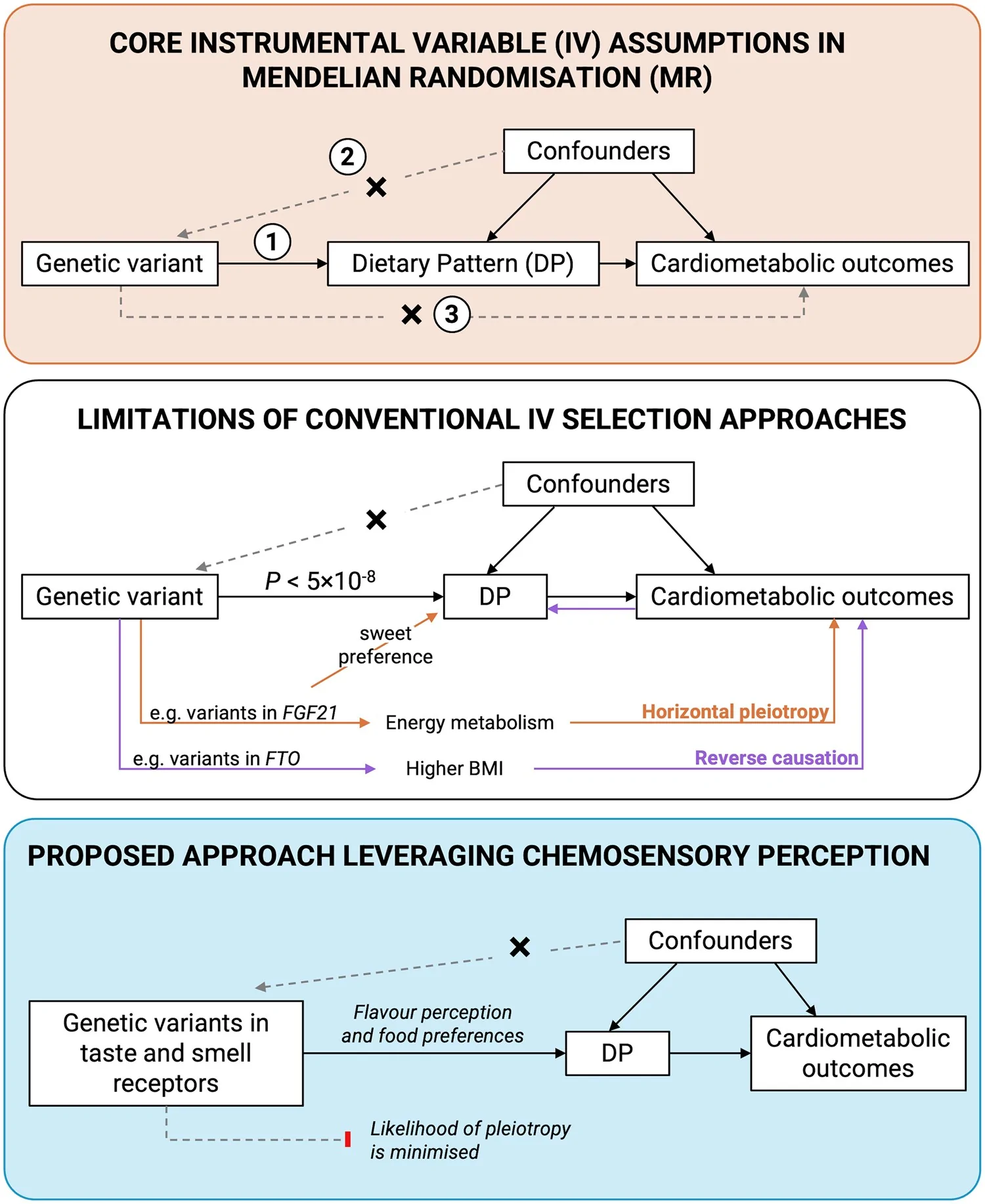
Comparison of instrument-selection strategies for DPs in MR: conventional significance-based methods versus biologically informed approaches based on chemosensory perception. Top panel shows the three core conditions that IVs should satisfy for MR to provide valid estimates of the causal effect, namely (i) relevance, (ii) independence, and (iii) exclusion–restriction. Middle panel illustrates the potential violations of the exclusion–restriction assumption and reverse causation if selecting IVs solely based on a significance threshold from dietary GWASs. Pathways labelled “Horizontal pleiotropy” (orange; *FGF21* example) illustrate pathways in which the genetic variant affects the outcome independently of DP. Pathways labelled “Reverse causation” (purple; *FTO* example) illustrate pathways in which the outcome influences exposure. Bottom panel presents a biologically informed approach using chemosensory-related variants to address such limitations. BMI, body mass index.

Diet is a major risk factor for cardiometabolic diseases, yet causal inference remains challenging. Observational epidemiology is susceptible to unmeasured confounding and reverse causation, while long-term dietary randomized–controlled trials (RCTs) face practical and ethical challenges [1]. Mendelian randomization (MR) offers an alternative by using genetic variants as instrumental variables (IVs) to proxy modifiable risk factors, such as diet [1–3]. As alleles are randomly allocated at meiosis, MR can be conceptualized as nature’s RCT, enabling causal inference from observational data. For valid causal estimates, three core assumptions must hold: (i) the genetic variants are robustly associated with the exposure, (ii) no confounders are associated with both the variant and the outcome, and (iii) the variant only (potentially) affects the outcome through the exposure, with no alternative pathways [4] (Fig. 1, top panel).

Most diet-related MR studies have examined single foods or nutrients [5, 6]. However, dietary exposures are typically compositional, where changes in one food imply changes in another in the opposite direction [6]. Genome-wide significant variants for specific dietary components may therefore capture correlated intakes rather than just the intended exposure [6], introducing horizontal pleiotropy. Dietary patterns (DPs), often derived by using principal component analysis (PCA), may better capture food combinations and substitutions in real-world diets [7]. Recent genome-wide association studies (GWASs) have identified genetic variants associated with such PCA-derived patterns [8–10].

However, DP GWAS-derived instruments raise methodological concerns. DP GWASs often identified variants at loci such as *FTO*, *APOE*, and *FGF21* [8–10], which have broad metabolic effects and may influence cardiometabolic outcomes independently of DP (Fig. 1, middle panel, horizontal pleiotropy pathway, orange arrows). Associations may also reflect reverse causation, as a diagnosis of cardiovascular disease can alter dietary behaviour towards healthier habits [9, 10], leading to misspecification of the exposure if these variants are used as IVs (Fig. 1, middle panel, reverse-causation pathway, purple arrows). Approaches such as Steiger filtering [11] or phenome-wide association studies (PheWAS) can help to identify variants with incorrect directionality or potentially pleiotropic associations [5]. Prioritizing variants with functional or biological relevance to the exposure trait over those chosen purely on statistical grounds may also reduce the risk of both horizontal pleiotropy and reverse causation [3, 5, 12].

Variants related to chemosensory perception offer one such biologically grounded option for dietary exposures [13]. Chemosensory receptor variants influence taste, smell, and chemesthetic perception through the receptor-mediated detection of food compounds, creating individual variability in flavour sensitivity that shapes food preference and choices [14, 15]. Receptor variants have been linked to specific dietary behaviours, including lower intake of vegetables [16], added salt [17], and red wine [17], ultimately shaping broader DPs. This biological specificity means that chemosensory-associated variants are less likely to influence cardiometabolic outcomes through non-dietary pathways, reducing the risk of horizontal pleiotropy and reverse causation (Fig. 1, bottom panel). Chemosensory receptor variants with consistent effects on food preferences across young and late adulthood have been used as IVs for specific food items in MR studies of cardiometabolic health [13].

This study applies MR to examine the causal effects of DPs on cardiometabolic outcomes while addressing these methodological challenges. In particular, cardiometabolic outcomes were selected, as their relationships with diet are well established and can provide a benchmark for validating the use of MR and biologically informed instruments for complex dietary exposures. Two complementary sets of instruments were used: (i) genome-wide significant (*P *< 5 × 10^−8^) variants from DP GWASs as primary IVs, with comprehensive IV filtering and sensitivity analyses to detect and control for potential confounding and horizontal pleiotropy; and (ii) chemosensory receptor variants as biologically informed instruments less likely to influence outcomes through non-dietary pathways.

## Methods

### Study design

**Figure 2:**
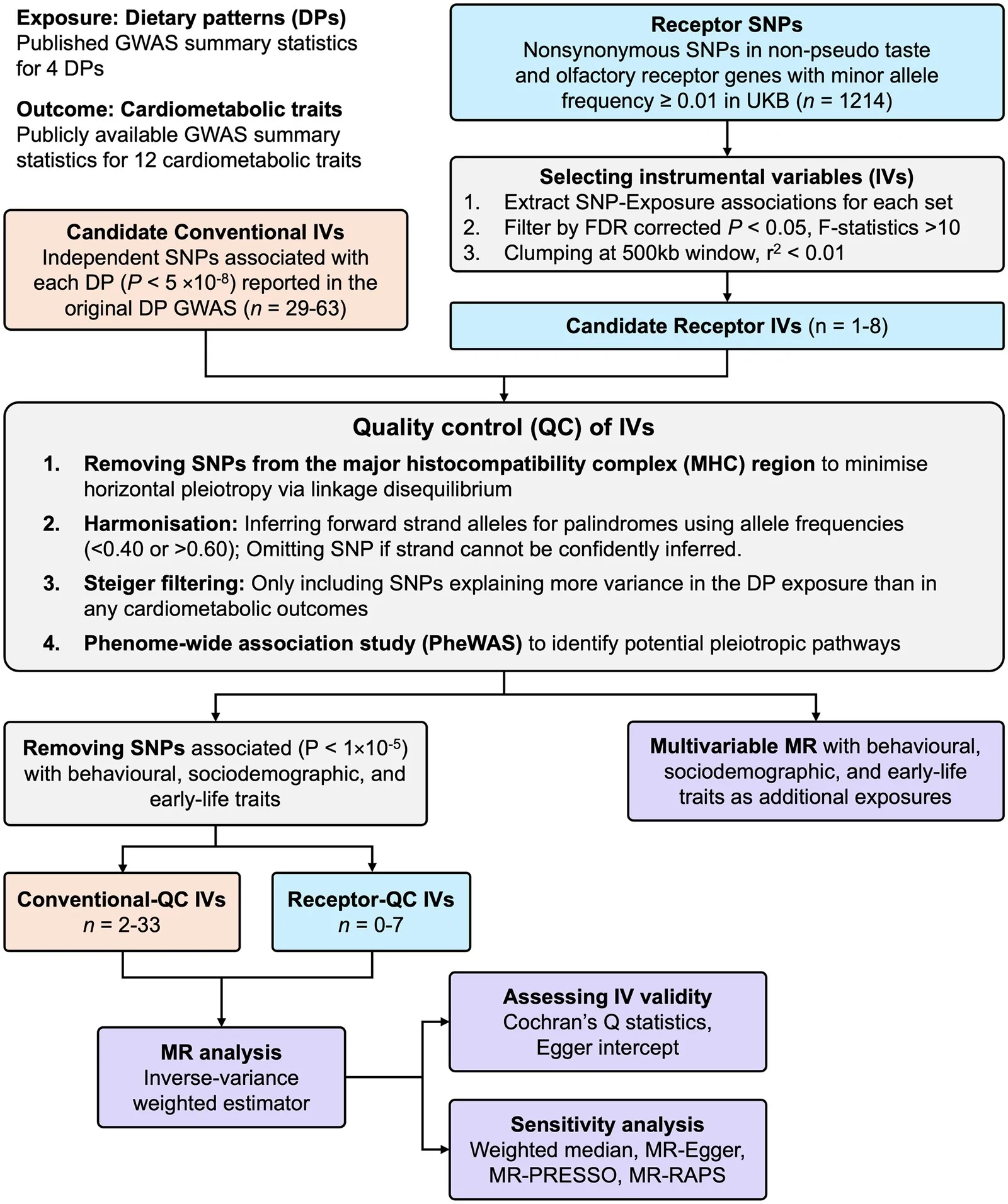
Study overview. Behavioural traits included smoking status, physical-activity levels, and sedentary time. Socio-economic traits included educational attainment, household income, housing tenure, and the Townsend Deprivation Index. Early-life traits included comparative body size at age 10 years. FDR, false discovery rate; MHC, major histocompatibility complex; MR-PRESSO, Mendelian randomization Pleiotropy RESidual Sum and Outlier; MR-RAPS, Mendelian randomization using a robust adjusted profile score; QC, quality control; SNP, single-nucleotide polymorphism; UKB, UK Biobank.

This study followed a two-sample MR framework to estimate the causal effects of DPs on cardiometabolic outcomes by using summary-level data from existing GWASs. The overall workflow is illustrated in Fig. 2. Reporting followed the STROBE-MR checklist [18].

### Data sources

Details for all exposure and outcome GWASs are summarized in Supplementary Table 1. Analyses were restricted to European ancestry, to minimize potential bias from population stratification.

GWAS summary statistics for DPs were obtained from three UK Biobank studies in which dietary intake was assessed by using a touchscreen questionnaire and DPs were derived by using PCA [8–10]. The characteristics of each DP, including their derivation and defining food components, are described in Table 1. DP scores reflect adherence to each pattern, with higher scores indicating greater intake of foods with positive loadings and lower intake of foods with negative loadings within that DP. However, as PCA-derived DPs are sensitive to the way in which dietary variables were processed and scaled within each study, a 1-SD increase in a DP does not correspond to a fixed absolute change in food intake and is not directly comparable across DPs.

**Table 1:** Description of DPs used as exposures in the present study.

DP	Description	DP derivation	Reference
Overall unhealthy (Unhealthy)	Lower intake of fish, fruit, and vegetables + Higher intake of coffee and alcohol + Higher intake of beef, pork, processed meat, lamb, and poultry + More added salt to diet	GWAS was conducted on 29 individual food items and genetic correlations between the items were estimated via bivariate LD score regression. PCA was applied to the genetic correlation matrix to derive ‘All PC’ (PC1) and GWAS was performed on individual ‘All PC’ scores	Pirastu *et al.* [8]
Overall healthy (Healthy)	Primarily defined by choosing wholemeal over white bread, along with higher intake of fruit, vegetables, oily fish, and water, and lower intake of processed meat	Diet-related questionnaire responses (35, including nested questions) were converted into 85 food-intake quantitative traits (FI-QTs) through ordinal ranking and binary encoding. PCA was then applied to the phenotypic correlation matrix of the 85 FI-QTs to derive PC scores and GWAS performed on individual PC scores	Cole *et al.* [9]
Meat-based	High intake of processed meat, poultry, beef, lamb, and pork. Orthogonal to the plant/fish-based diet	PCA applied on a phenotypic correlation matrix (questionnaire responses for fruit, vegetables, fish, and meat), with the Meat-based DP (‘DC1’) corresponding to PC1. GWAS then performed on individual ‘DC1’ scores	Niarchou *et al.* [10]
Plant/fish-based (Pescatarian)	High intake of raw and cooked vegetables, fresh and dried fruits, and oily and non-oily fish. Orthogonal to the meat-based diet	Corresponds to PC2 (‘DC2’) from the same PCA as above. GWAS performed on individual ‘DC2’ scores	Niarchou *et al.* [10]

DC, dietary component; FI-QT, food-intake quantitative trait; LD, linkage disequilibrium; PC, principal component.

Cardiometabolic outcomes included body mass index [19], coronary artery disease (CAD) [20], high-density lipoprotein cholesterol, low-density lipoprotein cholesterol, total cholesterol, total triglycerides [21], systolic blood pressure (SBP), diastolic blood pressure (DBP) [22], type 2 diabetes (T2D) [23], haemoglobin A1c (HbA1c), fasting glucose, and fasting insulin (FI) [24]. Where possible, outcome GWASs were selected from meta-analyses that excluded UK Biobank participants, although overlap remained for CAD, SBP, DBP, and T2D (40%, 45%, 45%, and 10%, respectively).

### Instrument selection and quality control

‘Conventional’ IVs were the independent, genome-wide significant variants reported in the source GWASs for each DP [8–10]. ‘Receptor’ IVs were sourced from 1214 non-synonymous single-nucleotide polymorphisms (SNPs) within non-pseudo taste and olfactory receptor genes with a minor allele frequency of ≥0.01 in UK Biobank identified by Hwang *et al.* [13], which were then screened for associations with each DP (false discovery rate-corrected *P *< .05; F-statistics > 10) and clumped at 500 kb and *r*^2^ < 0.01. SNPs within 1 Mb of the major histocompatibility complex (MHC) region were excluded due to extensive linkage disequilibrium in this region.

All candidate IVs underwent PheWAS to identify potential pleiotropy and reverse causation (full details are provided in Supplementary Section 1 and Supplementary Tables 2 and 3). Briefly, SNPs were screened for associations with potential confounders of diet–disease relationships, such as socio-economic factors. Flagged SNPs were either excluded from univariable MR or accounted for by using multivariable MR (MVMR) models [25, 26]. Directionality was assessed by using Steiger filtering [11], only retaining SNPs explaining more variance in the DP than in any of the 12 outcomes. All remaining variants constitute the post-quality control (post-QC) IV set.

For MVMR, five likely confounding traits were included alongside each DP: educational attainment, household income, physical activity, smoking status [27], and comparative body size at age 10 years [28] (Supplementary Table 1). Instrument strength was assessed via the Sanderson–Windmeijer conditional F‐statistics (MVMR R package v0.4) [29]. Details on exposure selection and covariance estimation are provided in Supplementary Section 1.

### Genetic correlation analysis

Bivariate Linkage Disequilibrium Score regression (v1.0.1) [30, 31] was used to estimate genetic correlations (*r*_g_) among the four DPs and the cardiometabolic outcomes. The multiple testing correction threshold was determined by using the effective number of independent tests (see Supplementary Section 1 and Supplementary Table 4). Accordingly, the Bonferroni-corrected significance threshold for the present study was *α* = 0.05/(3 × 11) = 1.52 × 10^−3^.

### MR

All MR analyses were conducted in R v4.4.2 using TwoSampleMR (v0.6.19) [11, 32] and ieugwasr (v1.0.4) [33]. Causal effects were estimated by using the inverse-variance-weighted (IVW) method. IV validity was assessed by using Cochran’s Q statistic for heterogeneity in causal-effect-size estimates and the MR–Egger intercept was used for directional pleiotropy [34].

### Sensitivity analyses

Complementary estimators with different underlying assumptions were used to assess the robustness of the primary IVW results. MR–Egger [34, 35] and the weighted median estimator [36] were used to obtain causal effect estimates that were more robust to invalid IVs. Where heterogeneity was detected, the Mendelian Randomization Pleiotropy RESidual Sum and Outlier (MR-PRESSO) test, implemented using the MRPRESSO package (v1.0) [37], was used to estimate causal effects with outliers removed. The MR robust adjusted profile score (MR-RAPS) method was used to evaluate the robustness of the estimates under potential weak instrument bias and balanced pleiotropy [38].

### Power calculations

Power was estimated by using the equations of Brion *et al.* [39] to determine the minimum detectable effect sizes at 80% power for *α* = 0.05 and *α* = 0.00152 (Bonferroni correction). For binary outcomes, alternative equations provided through an online power calculator (https://shiny.cnsgenomics.com/mRnd/) were used. As these equations were developed for one-sample MR, the power estimates (Supplementary Table 5) are for approximation only.

## Results

### SNP associations with DPs

From the original DP GWASs, we identified 16, 60, 29, and 63 Conventional IVs for the Unhealthy, Healthy, Meat-based, and Pescatarian DPs, respectively. Two Pescatarian DP SNPs were removed due to proximity to the MHC region. After the removal of SNPs associated with behavioural, socio-economic, or early-life traits (*P *< 1 × 10^−5^) and those failing Steiger filtering, post-QC Conventional IVs were 2, 22, 12, and 30, respectively, for the Unhealthy, Healthy, Meat-based, and Pescatarian DPs, with no overlapping IVs between DPs.

For Receptor IVs, initial counts were one, eight, one, and seven for the Unhealthy, Healthy, Meat-based, and Pescatarian DPs. No Receptor IVs were associated with the likely confounding traits. Instead, four Receptor IVs were excluded due to proximity to the MHC region and one Pescatarian IV failed Steiger filtering for SBP and DBP. Removal of these SNPs resulted in seven post-QC Receptor IVs for the Healthy DP, four for the Pescatarian DP, and none for the Unhealthy and Meat-based DPs remaining (Supplementary Table 6).

The associations of each IV with its respective DP and with each outcome, alongside variance explained, Steiger filtering, and PheWAS-based QC results, are reported in Supplementary Tables 7 and 8 for, respectively, Conventional and Receptor SNPs.

### Results from main analysis using conventional instruments

**Figure 3:**
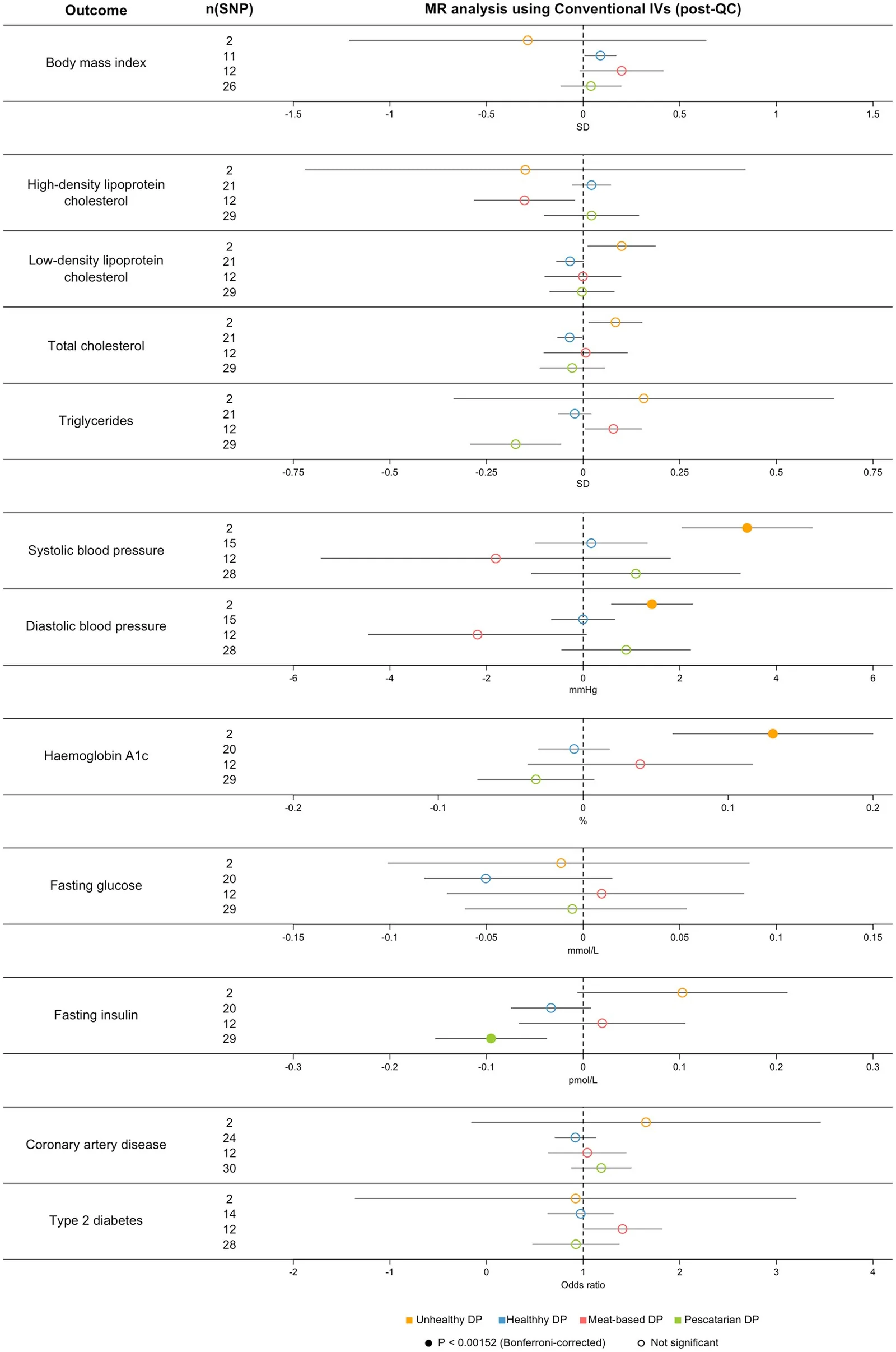
IVW estimates for the causal effects of the DPs on cardiometabolic outcomes using filtered Conventional instruments. Estimates represent the expected change in outcome for quantitative traits (in the units reported by the corresponding GWAS) or the expected odds of disease for binary outcomes per unit increase in principal-component-based DP. Error bars indicate 95% confidence intervals.

Using conventional IVs that passed QC filters (see ‘Methods’), four causal associations were observed at the Bonferroni-corrected threshold (*P *< 1.52 × 10^−3^). Each SD increase in the Unhealthy DP score was associated with higher SBP [*β*_IVW_ = 2.54 mmHg per SD, 95% confidence interval (CI) 1.53, 3.56; *P *= 8.64 × 10^−7^], DBP (*β*_IVW_ = 1.07 mmHg per SD, 95% CI 0.44, 1.70; *P *= 9.03 × 10^−4^), and HbA1c (*β*_IVW_ = 0.13% per SD, 95% CI 0.06, 0.20; *P *= 2.54 × 10^−4^), while each SD increase in Pescatarian DP scores was associated with reduced FI (*β*_IVW_ = −0.10 pmol/L per SD, 95% CI −0.15, −0.04; *P *= 1.19 × 10^−3^). The MR effect estimates for all DP–outcome pairs are shown in Fig. 3 and Supplementary Table 9.

No heterogeneity was detected for these four causal associations (Cochran’s Q *P *> .05), although heterogeneity was observed across DP–outcome pairs that yielded non-significant associations with IVW (Supplementary Table 10). Residual pleiotropy cannot be fully excluded despite the QC procedures applied, partly because PheWAS is constrained by available phenotypic data and may not capture all potential pleiotropic pathways. For most heterogeneity-positive associations, outlier IVs were identified in MR-PRESSO analyses. However, exclusion of these outliers, together with other sensitivity analyses, yielded estimates that were generally consistent with IVW in direction and size. Overall, these findings suggest that no substantial influence of residual pleiotropy was detected by the applied sensitivity analyses.

Interpretation of the Unhealthy DP findings warrants particular caution. Following QC, only two IVs remained. Substantial overlap was present between the DP and blood-pressure GWASs (45%), which may have increased susceptibility to weak instrument bias towards the observed associations. Heterogeneity was detected in five outcomes, with rs12568918 showing inconsistent directions of effect across cardiometabolic traits (Supplementary Table 6). This suggests potential horizontal pleiotropy through pathways independent of DP, despite the SNP passing Steiger filtering for directionality. The remaining IV, rs4410790, is strongly associated with caffeine consumption [40] and explained more variance in coffee intake than in the Unhealthy DP (*R*^2^ for coffee = 46.6%; *R*^2^ for Unhealthy DP = 7.1%) [8], suggesting that the observed associations with blood pressure and HbA1c likely reflect the effects of caffeine rather than broader DPs.

Directional pleiotropy could not be assessed for the Unhealthy DP associations, as fewer than three IVs were available, while the Pescatarian DP–FI association showed no evidence of directional pleiotropy (Egger intercept *P *> .05). Complete heterogeneity and pleiotropy statistics are provided in, respectively, Supplementary Tables 10 and 11.

### Results from sensitivity and MVMR analyses using conventional instruments

Sensitivity analyses using the weighted median estimator, MR–Egger, MR-PRESSO, MR-RAPS, and MVMR are presented, along with the primary IVW estimates, in Supplementary Table 9.

For the Unhealthy DP, MR-RAPS estimates were consistent with IVW inference, while other sensitivity estimators could not be applied due to the limited number of IVs. MVMR estimates were directionally consistent with the primary IVW results, but with wide CIs overlapping the null and evidence of weak instrument bias (conditional *F*_Unhealthy_ = 2.96; Supplementary Table 12).

For the Pescatarian DP–FI association, the weighted median and MR–Egger estimates were directionally consistent with IVW, but the CIs overlapped with the null. MR-PRESSO did not detect any outlier IVs. MR-RAPS (*P *= 7.39 × 10^−3^) and MVMR (*P *= 3.02 × 10^−2^) replicated this association at nominal significance, with conditional F-statistics indicating weak instruments (*F*_Pescatarian_ = 2.90; for selected traits = range 1.84–9.67; see Supplementary Table 12). As such, the MVMR estimates were interpreted cautiously and only used to assess the consistency of effect direction with the primary IVW analysis.

### Results from main analysis using receptor instruments

**Figure 4:**
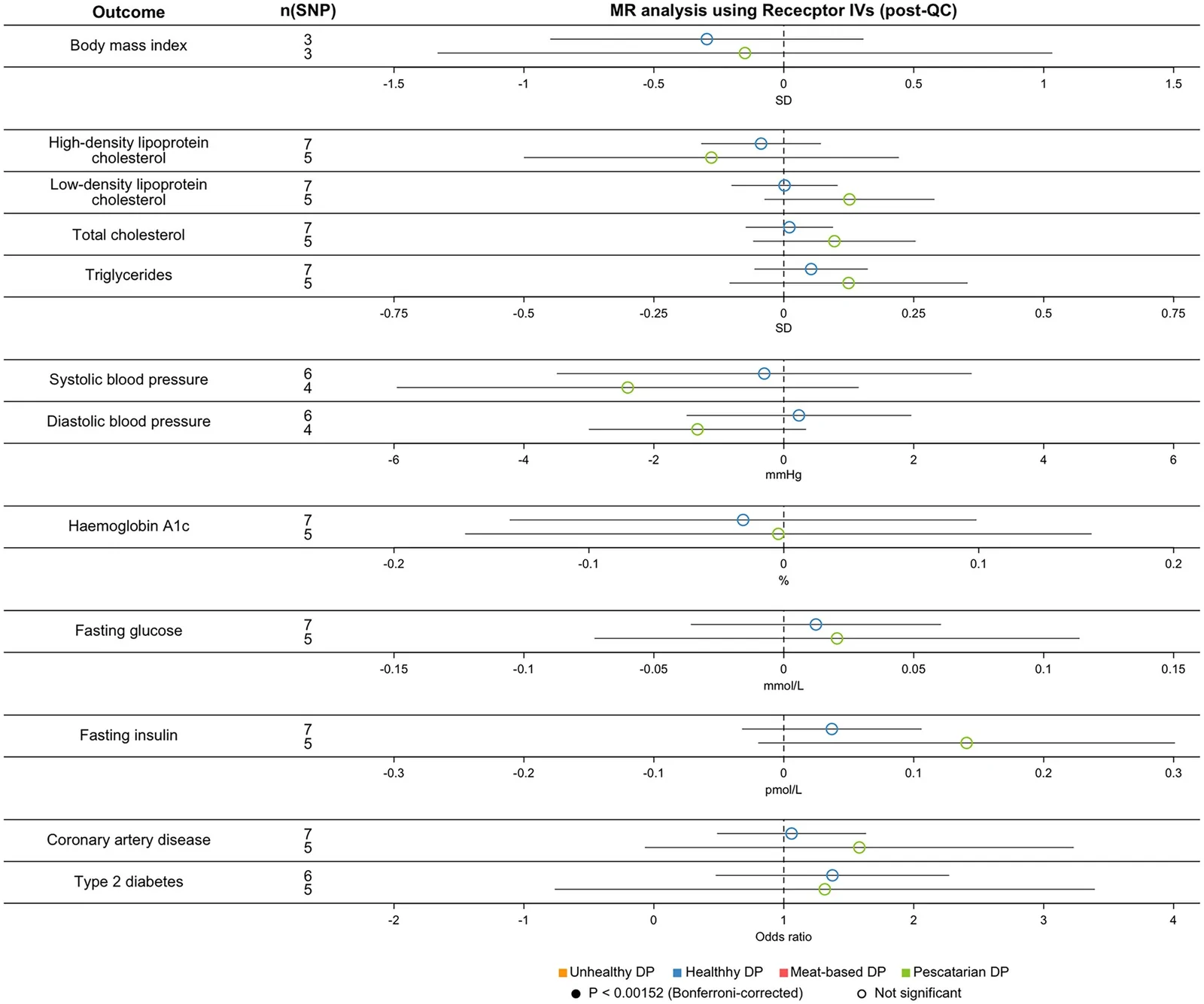
IVW estimates for the causal effects of the DPs on cardiometabolic outcomes using Receptor instruments. Estimates represent the expected change in outcome for quantitative traits (in the units reported by the corresponding GWAS) or the expected odds of disease for binary outcomes per unit increase in the principal-component-based DP. Error bars indicate 95% confidence intervals. No Receptor instruments remained for the Meat-based and Unhealthy DPs after quality control.

MR estimates using Receptor IVs are provided in Supplementary Table 13. Upon MHC exclusions, no IVs remained for the Unhealthy and Meat-based DPs. For the Healthy and Pescatarian DPs, all estimates overlapped with the null (Fig. 4), with heterogeneity in the causal estimates and directional pleiotropy detected (*P *< .05) for some associations (Supplementary Tables 10 and 11).

### Results from sensitivity analysis using receptor instruments

All weighted median, MR–Egger, and MR-RAPS estimates overlapped with the null, except for a suggestive association between the Healthy DP and reduced SBP (weighted median *P *= 4.05 × 10^−2^). However, this finding may reflect weak instrument bias towards the observed association, particularly in the presence of sample overlap between the DP and SBP GWASs. MR-PRESSO either did not detect any outliers or the outlier-corrected estimates overlapped with the null.

## Discussion

This study used MR to investigate the causal effects of DPs on cardiometabolic outcomes, emphasizing rigorous IV selection and attempting to compare biologically informed against conventional, significance-based IV selection strategies. Overall, evidence for the independent causal effects of broad DPs on cardiometabolic outcomes was limited to a protective effect of the Pescatarian DP on reducing FI. The apparent associations of the Unhealthy DP with blood pressure and HbA1c should be interpreted cautiously; only two IVs remained, one of which was more strongly associated with coffee intake than with the DP itself. In addition, sample overlap between the DP and blood-pressure GWASs may have increased susceptibility to weak instrument bias towards the observed phenotypic associations [41, 42]. Chemosensory Receptor IVs yielded null findings across DP–outcome pairs, likely reflecting insufficient statistical power.

The protective effect of a genetically predicted Pescatarian DP on FI aligns with observational [43, 44] and experimental [45] evidence for Mediterranean-style diets, in which oily fish, vegetables, fruits, and minimal processed meat are emphasized. Fish consumption in particular may improve insulin sensitivity via omega-3 fatty acids reducing the lipid-induced impairment of insulin signalling [43], while dietary fibre has been associated with improved FI in RCTs, potentially through the gut microbiota [46]. However, FI generally varied by tens of picomoles per litre [24], indicating that the MR effect estimate of −0.10 pmol/L per SD of the Pescatarian DP score is very small relative to the population-level variation in FI. Consistently with this small effect size, null findings for downstream outcomes (HbA1c, T2D) suggest that DP alone, when isolated from other risk factors through MR, may be insufficient to produce clinically detectable changes in glycaemic disease outcomes, consistent with diet functioning as one causal risk factor within a multifactorial disease process.

The null findings for chemosensory Receptor IVs likely reflect both statistical and biological limitations. Biologically informed IVs may offer higher specificity, but typically explain less variance in the exposure compared with a larger collection of genome-wide-significant SNPs [3]. The effects of chemosensory variants appear to be stronger for individual food items than for composite DPs [13], with their signals diluted when aggregated across multiple foods. This reduced explained variance, coupled with fewer IVs, meant that the chemosensory approach had low statistical power in detecting small-to-moderate causal effects, if they exist [39]. ‘Winner’s curse’ [47] may further bias effect estimates towards the null. Future research should prioritize identifying additional biologically relevant variants with stronger effects on DPs or developing statistical methods that aggregate pathway-specific IVs for individual food items (e.g. chemosensory receptor variants for specific food items, lactase persistence for dairy intake) to model DPs, thereby retaining biological specificity.

A broader challenge lies in translating MR findings by using PCA-derived DPs into practical recommendations, as they reflect relative intake combinations rather than absolute quantities. Guideline-based indices with absolute intake targets, such as the Healthy Eating Index [48], may offer clearer translational value, as scoring is based on real-world food-group units, allowing the identification of specific intake shortfalls. Unlike PCA-derived patterns, in which loadings and scale shift across study populations, fixed scoring frameworks help to retain interpretability across different contexts. Wider adoption of such indices in dietary MR requires GWAS summary statistics for specific a priori patterns, while PCA remains valuable for studying associations between existing DPs and health outcomes.

## Conclusion

This study examined the causal effects of DPs on cardiometabolic health by using two-sample MR, with findings limited to support a protective association between a Pescatarian DP and FI. Although multiple strategies were applied to mitigate horizontal pleiotropy, including IV filtering and multivariable modelling, residual pleiotropy remains a general limitation of MR with complex dietary exposures. Chemosensory receptor variants were explored as biologically informed instruments, though the insufficient variance explained in composite DPs limited statistical power. Future work should prioritize GWASs of guideline-based diet-quality scores and methods that aggregate pathway-specific variants across food items to model overall DPs.

## Ethics approval

This study primarily used publicly available GWAS summary statistics. UK Biobank individual-level data were accessed under application 53641 for estimating phenotypic covariances between exposures in MVMR. UK Biobank holds ethical approval from the North West Multi-Centre Research Committee (21/NW/0157).

## Supplementary Material

dyag186_Supplementary_Data

## Data Availability

GWAS summary statistics of the Meat-based and Pescatarian DPs were obtained from the authors of Niarchou *et al.* [10]. All other GWAS summary statistics used in this study are publicly available from the GWAS Catalog (https://www.ebi.ac.uk/gwas/), the OpenGWAS website (https://gwas.mrcieu.ac.uk), and the DIAGRAM Consortium (http://www.diagram-consortium.org/downloads.html), with details of the data sources provided in Supplementary Table S1. UK Biobank individual-level data are available to approved researchers from UK Biobank (http://www.ukbiobank.ac.uk/).
