# Supplementary material for: Comparing genome-wide significant and chemosensory variants as instruments for dietary patterns in Mendelian randomization
Source: Int J Epidemiol. 2026 Sep 9;55(5):dyag186. doi: 10.1093/ije/dyag186 (PMC13557626; doi:10.1093/ije/dyag186)
Supplement: Supplementary file 1 [file dyag186_supplementary_data.zip › ije-2026-03-0370-File007.docx]

# Supplementary Contents

| **Supplementary Section 1:** Instrument screening and analytical methods | Page 2-4 |
| --- | --- |
| **Supplementary Table S1:** GWAS data sources for exposures, outcomes, and traits used in multivariable MR | Provided in separate Excel file |
| **Supplementary Table S2:** PheWAS associations with sociodemographic, lifestyle, and early-life traits |  |
| **Supplementary Table S3:** Proxy SNPs |  |
| **Supplementary Table S4:** Overlapping instruments and genetic correlations between dietary patterns and cardiometabolic outcomes |  |
| **Supplementary Table S5:** Power estimation |  |
| **Supplementary Table S6:** Instrumental variables across four dietary pattern exposures |  |
| **Supplementary Table S7:** SNP associations with each dietary pattern and cardiometabolic outcome for Conventional instruments |  |
| **Supplementary Table S8:** SNP associations with each dietary pattern and cardiometabolic outcome for Receptor instruments |  |
| **Supplementary Table S9:** MR effect estimates using Conventional IVs |  |
| **Supplementary Table S10:** Cochran's Q statistics for heterogeneity assessment of IVW estimates |  |
| **Supplementary Table S11:** MR-Egger intercepts for directional pleiotropy assessment |  |
| **Supplementary Table S12:** Conditional F-statistics for multivariable Mendelian randomisation analyses |  |
| **Supplementary Table S13:** MR effect estimates using Receptor IVs |  |

# Supplementary Section 1: Instrument screening and analytical methods

## **PheWAS-based screening and pleiotropy assessment of instruments**

All candidate instrumental variables (IVs) underwent a two-stage phenome-wide association study (PheWAS) using ieugwasr v1.0.2 [1], scanning for associations across OpenGWAS (accessed 2 April 2025) [2,3]. Associations were considered at *P* < 1×10^-5^, approximating a Bonferroni-corrected threshold (0.05/4358 traits = 1.15×10^-5^) previously used by Cole et al. [4] based on the number of traits in the UK Biobank [2,3]. This correction is inherently conservative as a proportion of the GWAS queried involved overlapping individuals and correlated traits.

In Stage 1 (*a priori* sources of bias), SNPs were screened for associations with traits that could violate the exclusion-restriction criteria due to confounding, including early-life traits (e.g., birth outcomes, childhood anthropometry [age <18 years]) and adult behavioural and socioeconomic traits commonly considered in observational nutritional epidemiology: socioeconomic status (SES) (including income, housing tenure, number of vehicles, Townsend deprivation index), educational attainment, alcohol consumption, physical activity, and sedentary time **(Supplementary Table S2**). Flagged SNPs were either: (1) removed from univariable MR; or (2) adjusted for using multivariable MR (MVMR) models by incorporating these traits as additional exposures [5,6].

Stage 2 (exploratory) reviewed remaining PheWAS associations for additional pleiotropic pathways. Adult anthropometric and clinical traits were not treated as sources of bias, as these may lie downstream of DPs. Instead, directionality was addressed using Steiger filtering [7], retaining only SNPs explaining more variance in the DP than in any of the 12 outcomes.

Palindromic IVs were aligned using allele frequencies, where variants with allele frequencies between 0.40 and 0.60 were treated as ambiguous and omitted from all MR analyses. IVs absent from an outcome dataset were replaced with proxies (r^2^ > 0.8 in Europeans) identified via ieugwasr for outcomes available in OpenGWAS [2,3], LDproxy [8], followed by HaploReg v4.2 if still unavailable [9]. Manually identified proxies are listed in **Supplementary** **Table S3**.

## **Selection of covariates for multivariable MR (MVMR)**

Covariates included in MVMR were selected from domains identified in Stage 1 PheWAS screening as potential sources of confounding. To minimise collinearity, one representative trait was selected from each domain (e.g., household income, Townsend deprivation index, and housing tenure all representing SES), by prioritising GWAS conducted in the UK Biobank and had the greatest overlap between its instruments and DP IVs. The final model included five traits alongside each DP: educational attainment, household income, physical activity, smoking status [10], and comparative body size at age 10 [11] (**Supplementary** **Table S1**). Each covariate was treated as a potential confounder of the relationship between dietary patterns and cardiometabolic outcomes. In particular, childhood body size was included to account for genetic influences on early-life adiposity that may: (i) influence subsequent dietary behaviours and food selection patterns; and (ii) affect adult cardiometabolic outcomes through adiposity-related pathways. Thus, childhood body size was modelled as a pre-exposure confounder rather than a mediator of adult dietary effects.

***Estimation of phenotypic covariance and instrument strength in MVMR***

Instrument strength in MVMR was assessed via the Sanderson-Windmeijer conditional F‐statistics (MVMR R package v0.4) [12]. Phenotypic covariances among exposures in each MVMR model were estimated using UK Biobank individual-level data (*n* = 275 950 unrelated participants of European ancestry). DP scores were reconstructed following the original GWAS methods [8–10]. For the Unhealthy DP, defined using genetic correlations between intakes of individual food items, PCA was performed on a genetic correlation matrix reconstructed from the published pairwise genetic correlation estimates [13]. Non-dietary traits were also coded and adjusted per their respective GWAS protocols [10,11].

***Genetic correlation analysis***

Bivariate Linkage Disequilibrium Score (LDSC) regression (v1.0.1) [14,15] was used to estimate genetic correlations (r_g_) among the four DPs and the cardiometabolic outcomes (**Supplementary Table S4**). SNPs were restricted to the HapMap3 reference panel and excluded for MAF < 0.01, reference allele mismatches, strand ambiguity, or (where available) imputation INFO < 0.9. LD scores were calculated using the 1000 Genomes Phase 3 European reference panel.

***Multiple testing correction***

The effective number of independent tests was determined using matSpD [16] (<https://research.qut.edu.au/sgel/software/matspd-local-version/>) based on the genetic correlation matrices. The four DPs represented three independent exposures. Outcomes were grouped by consortium and analysed in blocks to account for sample overlap, yielding an effective number of 11 independent outcomes. Accordingly, the Bonferroni-corrected significance threshold for the present study was *α* = 0.05/(3×11) = 1.52×10^-3^.

**References**

1.	Hemani G, Elsworth B, Palmer T *et al.* *Ieugwasr: Interface to the ‘OpenGWAS’ Database API.*, version 1.0.2. 2024. https://github.com/MRCIEU/ieugwasr. (2 April 2025, date last accessed)

2.	Elsworth B, Lyon M, Alexander T *et al.* The MRC IEU OpenGWAS data infrastructure. *bioRxiv* published online 2020. https://doi.org/10.1101/2020.08.10.244293.

3.	Hemani G, Zheng J, Elsworth B *et al.* The MR-Base platform supports systematic causal inference across the human phenome. *eLife* 2018;**7**:e34408. https://doi.org/10.7554/eLife.34408.

4.	Cole JB, Florez JC, Hirschhorn JN. Comprehensive genomic analysis of dietary habits in UK Biobank identifies hundreds of genetic associations. *Nat Commun* 2020;**11**(1):1467. https://doi.org/10.1038/s41467-020-15193-0.

5.	Sanderson E, Davey Smith G, Windmeijer F *et al.* An examination of multivariable Mendelian randomization in the single-sample and two-sample summary data settings. *Int J Epidemiol* 2019;**48**(3):713–27. https://doi.org/10.1093/ije/dyy262.

6.	Burgess S, Thompson SG. Multivariable Mendelian Randomization: The Use of Pleiotropic Genetic Variants to Estimate Causal Effects. *Am J Epidemiol* 2015;**181**(4):251–60. https://doi.org/10.1093/aje/kwu283.

7.	Hemani G, Tilling K, Davey Smith G. Orienting the causal relationship between imprecisely measured traits using GWAS summary data. *PLOS Genet* 2017;**13**(11):e1007081. https://doi.org/10.1371/journal.pgen.1007081.

8.	Machiela MJ, Chanock SJ. LDlink: a web-based application for exploring population-specific haplotype structure and linking correlated alleles of possible functional variants. *Bioinformatics* 2015;**31**(21):3555–7. https://doi.org/10.1093/bioinformatics/btv402.

9.	Ward LD, Kellis M. HaploReg: a resource for exploring chromatin states, conservation, and regulatory motif alterations within sets of genetically linked variants. *Nucleic Acids Res* 2012;**40**(D1):D930–4. https://doi.org/10.1093/nar/gkr917.

10.	Elsworth B, Mitchell R, Mitchell R *et al.* MRC IEU UK Biobank GWAS pipeline version 2. University of Bristol dataset, 2019. University of Bristol dataset. https://doi.org/10.5523/BRIS.PNOAT8CXO0U52P6YNFAEKEIGI.

11.	Richardson TG, Sanderson E, Elsworth B *et al.* Use of genetic variation to separate the effects of early and later life adiposity on disease risk: mendelian randomisation study. *BMJ* 6 May 2020:m1203. https://doi.org/10.1136/bmj.m1203.

12.	Sanderson E, Spiller W, Bowden J. Testing and correcting for weak and pleiotropic instruments in two‐sample multivariable Mendelian randomization. *Stat Med* 2021;**40**(25):5434–52. https://doi.org/10.1002/sim.9133.

13.	Pirastu N, McDonnell C, Grzeszkowiak EJ *et al.* Using genetic variation to disentangle the complex relationship between food intake and health outcomes. *PLOS Genet* 2022;**18**(6):e1010162. https://doi.org/10.1371/journal.pgen.1010162.

14.	Schizophrenia Working Group of the Psychiatric Genomics Consortium, Bulik-Sullivan BK, Loh PR *et al.* LD Score regression distinguishes confounding from polygenicity in genome-wide association studies. *Nat Genet* 2015;**47**(3):291–5. https://doi.org/10.1038/ng.3211.

15.	ReproGen Consortium, Psychiatric Genomics Consortium, Genetic Consortium for Anorexia Nervosa of the Wellcome Trust Case Control Consortium 3 *et al.* An atlas of genetic correlations across human diseases and traits. *Nat Genet* 2015;**47**(11):1236–41. https://doi.org/10.1038/ng.3406.

16.	Nyholt DR. A Simple Correction for Multiple Testing for Single-Nucleotide Polymorphisms in Linkage Disequilibrium with Each Other. *Am J Hum Genet* 2004;**74**(4):765–9. https://doi.org/10.1086/383251.
